# Supplementary figures and images for: Analysis of microbiota in elderly patients with Acute Cerebral Infarction
Source: PeerJ. 2019 Jun 12;7:e6928. doi: 10.7717/peerj.6928 (PMC6571007; doi:10.7717/peerj.6928)

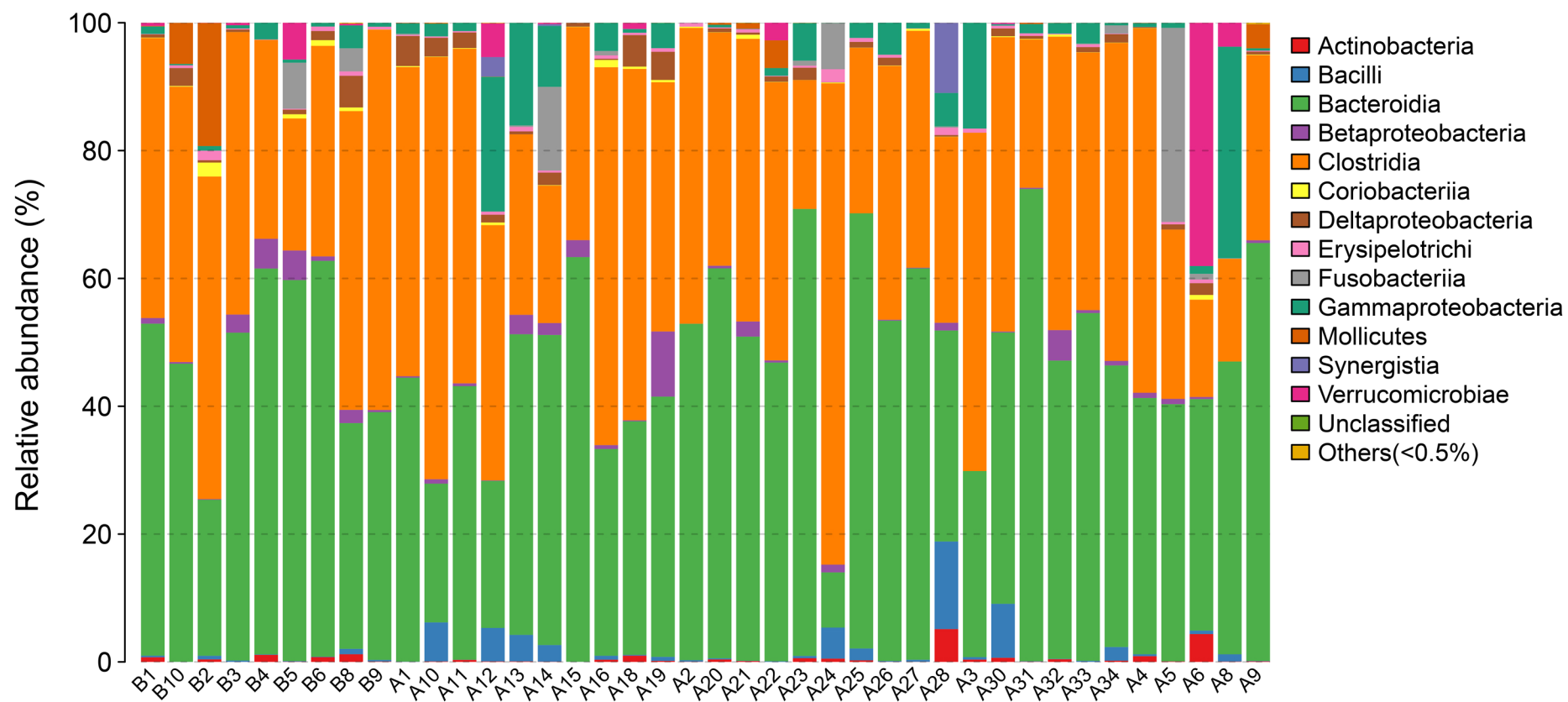

Supplement: Figure S1 — The relative abundance of each Class in the intestinal gut in each sample from Acute Cerebral Infarction patients and controls (A indicates patients and B indicates healthy people). [file peerj-07-6928-s002.pdf]

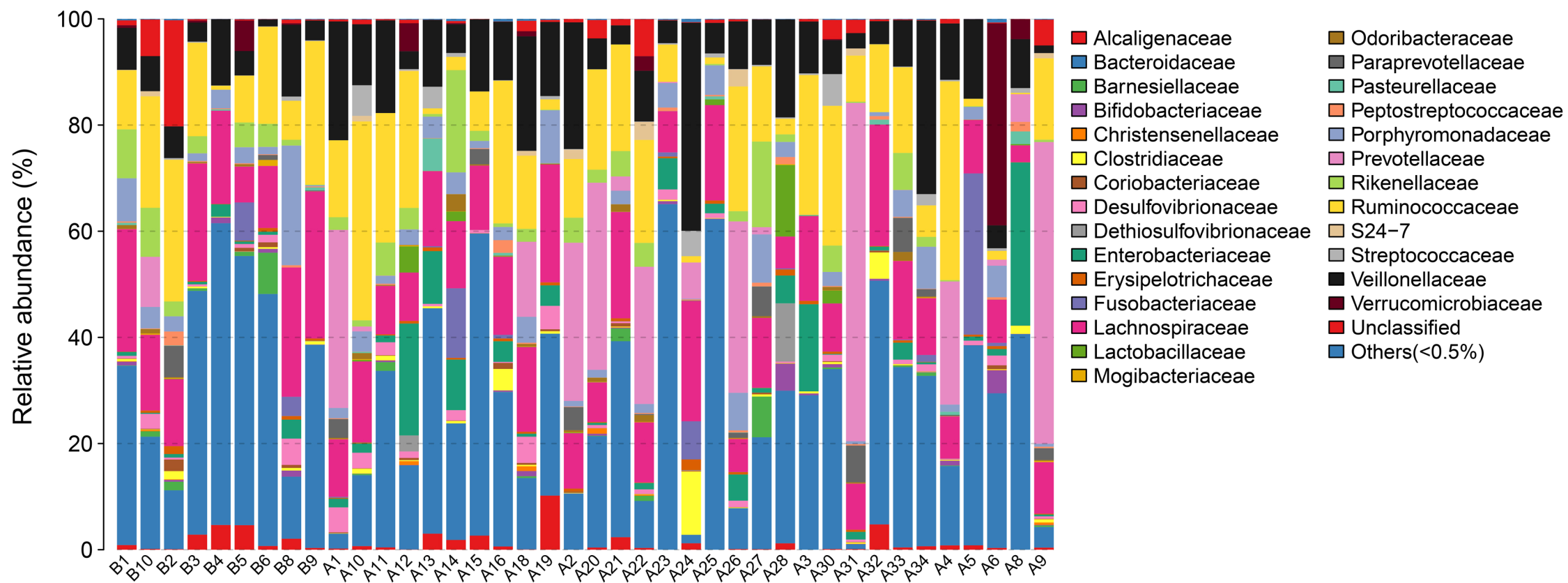

Supplement: Figure S2 — The relative abundance of each Family in the intestinal gut in each sample from Acute CerebralInfarction patients and controls (A indicates patients and B indicates healthy people). [file peerj-07-6928-s003.pdf]

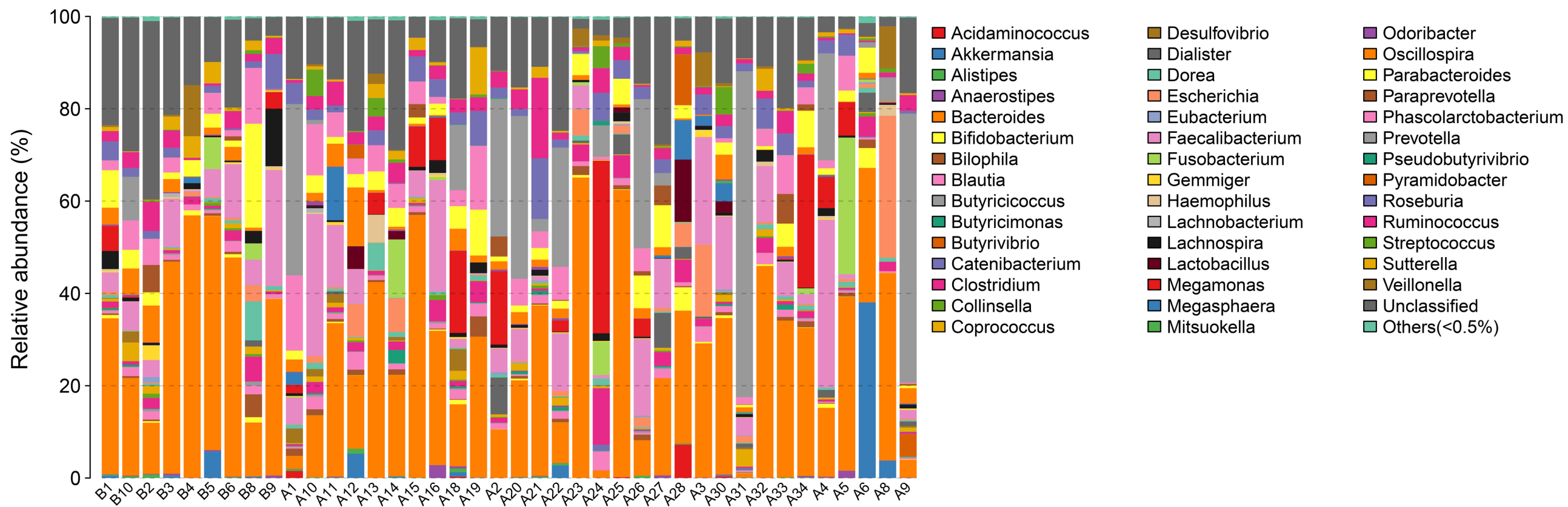

Supplement: Figure S3 — The relative abundance of each Genus in the intestinal gut in each sample from Acute CerebralInfarction patients and controls (A indicates patients and B indicates healthy people). [file peerj-07-6928-s004.pdf]

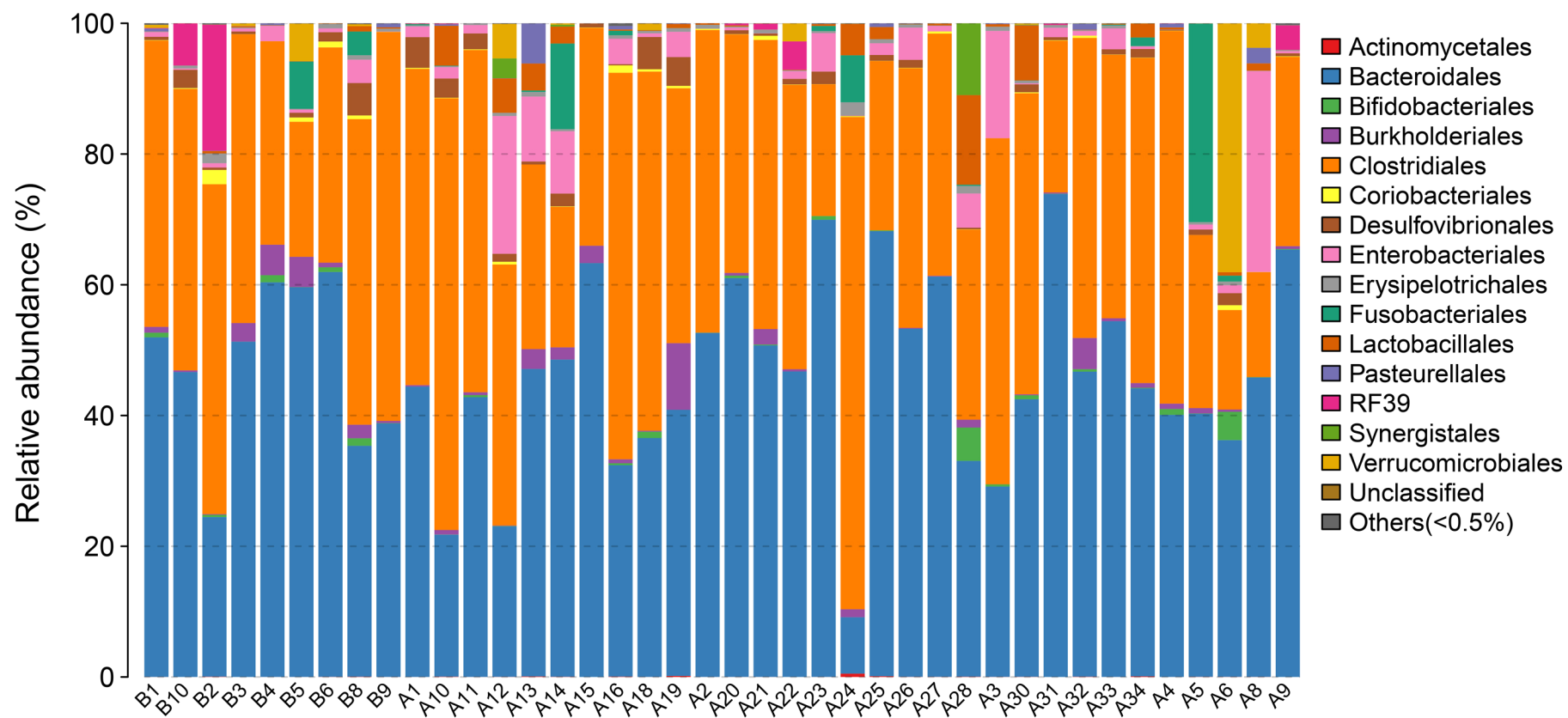

Supplement: Figure S4 — The relative abundance of each Order in the intestinal gut in each sample from Acute CerebralInfarction patients and controls (A indicates patients and B indicates healthy people). [file peerj-07-6928-s005.pdf]

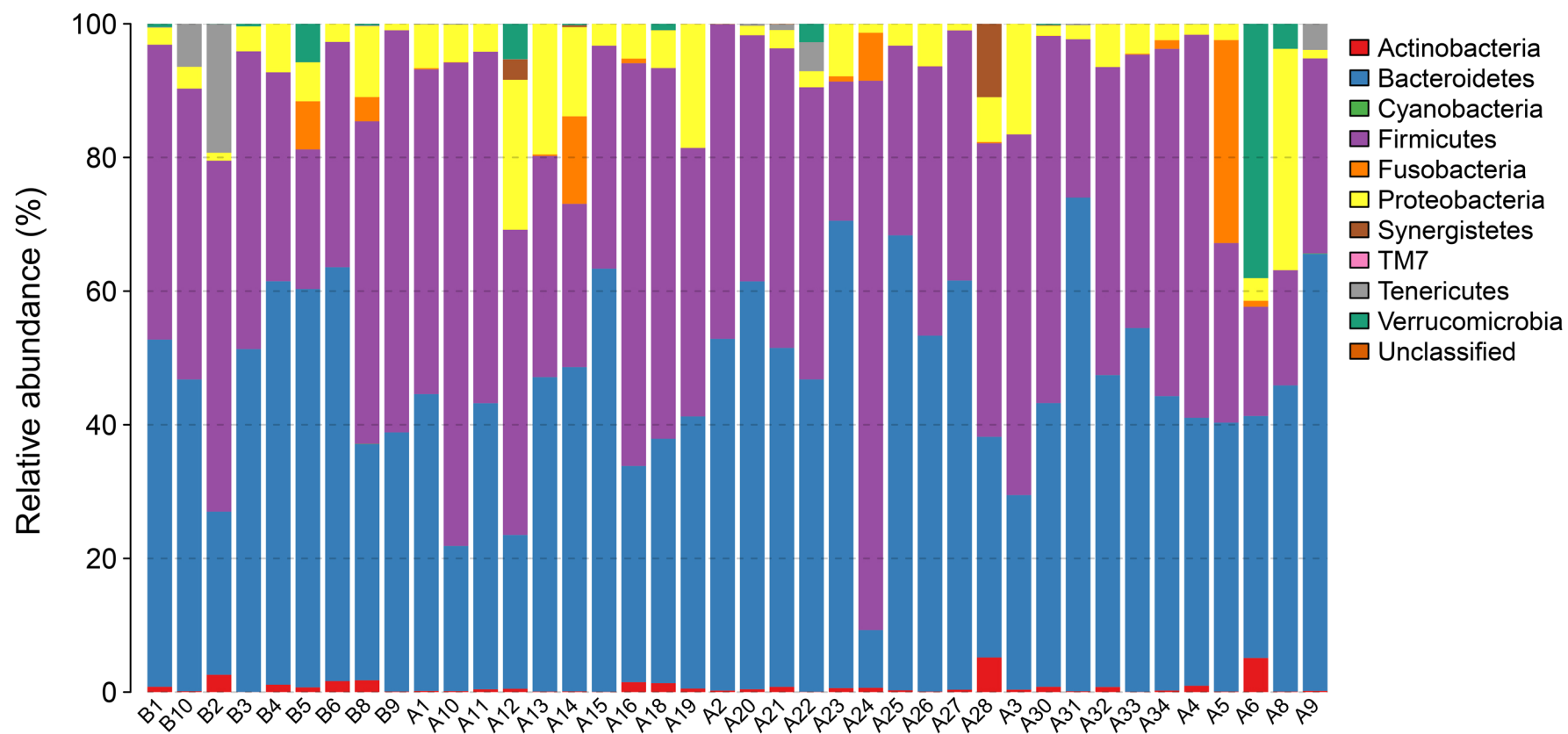

Supplement: Figure S5 — The relative abundance of each Phylum in the intestinal gut in each sample from Acute CerebralInfarction patients and controls (A indicates patients and B indicates healthy people). [file peerj-07-6928-s006.pdf]
